# VTE Risk Assessment Compliance in an Acute Psychiatric Inpatient Ward in Livewell Southwest

**DOI:** 10.1192/bjo.2026.11358

**Published:** 2026-06-30

**Authors:** Swanzil Chaudhary, Twisha Rohan, Malik Noor, Vicky Romback

**Affiliations:** 1 Livewell Southwest, Plymouth, United Kingdom; 2 University Hospital Plymouth, Plymouth, United Kingdom

## Abstract

**Aims:**

Venous thromboembolism (VTE) is a preventable cause of morbidity and mortality in hospitalised patients (Source: NHS England). Psychiatric inpatients are at an increased risk due to factors unique to our patient subset, such as reduced mobility, antipsychotic medication use, and illness-related behavioural changes including poor food/fluid intake, catatonia, being post-partum etc. Despite the NICE guidelines recommending VTE risk assessment including assessing psychiatric-specific risk factors for all hospital admissions, compliance within mental health inpatient settings remains variable. There also seems to be a focus on medical/surgical risk factors, and psychiatric-specific risk factors may get lost in this assessment. The aim of our QI Project is to evaluate compliance with national guidance on VTE risk assessment documentation on admission, and to identify potential gaps in assessment among patients with psychiatric-specific VTE risk factors.

**Methods:**

A retrospective review of inpatient records at the Glenbourne Unit in Plymouth,which is an acute psychiatric inpatient unit with a General Adult Male and Female ward, and an Old Age ward. Data were collected from a sample of 47 inpatients in September 2025.

The primary outcome was clear documentation of VTE risk assessment on admission proforma of the Trust, recorded as ‘Yes’ or ‘No’, alongside brief clinical reasoning for prescribing or not deciding to prescribe VTE prophylaxis. Data collected included patient demographics, mobility status, antipsychotic use on admission, presence of psychiatric-specific VTE risk factors (such as antipsychotic medication use, reduced oral intake, and psychomotor retardation or catatonia), and also existing medical conditions or previous history of VTE.

Compliance was assessed against NICE guideline NG89, which recommends VTE risk assessment on admission, reassessment within 24 hours, and when clinical circumstances change. The remits of our audit were VTE assessment on admission and within 24 hours.

**Results:**

The mean patient age was 43.9 years (range of 18–84years), with 25.5% aged over 60 years (hence at an increased risk of VTE). Clear documentation of VTE risk assessment on admission was seen in only 40% of records, while 51% were marked as 'not applicable'.

Among patients with more than one psychiatric risk factor (n=39), 59% lacked clear documentation. Of those prescribed antipsychotic medication (n=35), 51% had no clear record of VTE risk consideration.

Documentation rates were higher in older adults (66%) compared to working-age adults (46%). Patients who were immobile had higher rates of VTE risk documentation (62%) than mobile patients (48.7%).

Documentation rates were noted to be lower amongst patients receiving antipsychotic medication and those with reduced mobility on admission.

**Conclusion:**

Compliance with admission VTE risk assessment documentation in the Acute psychiatric inpatient unit was below the expected standard, including in patients with recognised psychiatric-specific risk factors. This highlights a need for improved awareness and to follow a structured assessment process. We believe that a number of factors might be at play for this, and a multi-step approach is essential.

We are currently due to start an intervention following the findings, to disseminate information, teaching sessions and presentation of our findings at a trust-level meeting so that this may trickle down into better compliance to the guidelines. We do have a built in VTE Risk Assessment Proforma on Systm One software, but this seems to be seldom used. Also, there needs to be more awareness to robustly consider psychiatric risk factors like we discussed above, to be considered for initial assessment of VTE risk.

